# Ebola: Improving the Design of Protective Clothing for Emergency Workers Allows Them to Better Cope with Heat Stress and Help to Contain the Epidemic

**DOI:** 10.1093/annhyg/mev003

**Published:** 2015-02-11

**Authors:** Kalev Kuklane, Karin Lundgren, Chuansi Gao, Jakob Löndahl, Elisabeth Dalholm Hornyanszky, Per-Olof Östergren, Per Becker, Marcella C. Samuels, Pernille Gooch, Catharina Sternudd, Maria Albin, Tahir Taj, Ebba Malmqvist, Erik Swietlicki, Lennart Olsson, Kenneth Persson, Johanna Alkan Olsson, Tord Kjellstrom

**Affiliations:** 1.Ergonomics and Aerosol Technology, Design Sciences, Lund University, PO Box 118, 22100 Lund, Sweden E-mail: kalev.kuklane@design.lth.se; 2.Social Medicine and Global Health, Lund University, PO Box 118, 22100 Lund, Sweden; 3.Centre for Societal Resilience, Lund University, PO Box 118, 22100 Lund, Sweden; 4.Human Ecology, Lund University, PO Box 118, 22100 Lund, Sweden; 5.Architecture and Build Environment, Lund University, PO Box 118, 22100 Lund, Sweden; 6.Occupational and Environmental Medicine, Lund University, PO Box 118, 22100 Lund, Sweden; 7.Nuclear Physics, Lund University, PO Box 118, 22100 Lund, Sweden; 8.Lund University Center for Sustainability Science, Lund University, PO Box 118, 22100 Lund, Sweden; 9.Water Resources Engineering, Lund University, PO Box 118, 22100 Lund, Sweden; 10.Centre for Environmental and Climate Research, Lund University, PO Box 118, 22100 Lund, Sweden; 11.Center for Work Environment and Leadership, Lund University, PO Box 118, 22100 Lund, Sweden; 12.Health and Environment International Trust, 168 Stafford Drive, Mapua, 7005 New Zealand; 13.Pufendorf Institute, Lund University, PO Box 118, 22100 Lund, Sweden

It is a complex task to find optimal protective clothing to prevent the spread of Ebola virus disease (Martin-Moreno *et al.*, 2014; Ryschon, 2014). The fear of getting infected is an obstacle for recruiting healthcare workers. In addition, the current design of protective clothing might curtail their working capacity severely in the hot and humid climate of West Africa and, in addition, paradoxically increase the risk of infection. Emergency work in full protective clothing including respiratory mask may lead to extreme heat stress in the hot climates resulting in shortened work time, dehydration, reduced professional judgement, and exhaustion. This increases risk of infection of health stuff (WHO, 2014).

In Monrovia, Liberia, daytime maximum temperatures in the end of the year often reach 30–31°C, and the temperatures will be higher January to May, the hot season (Kjellstrom et al., 2014; http://climatechip.org/). In order to manage this heat stress, the workers need breaks (Kjellstrom *et al.*, 2009). This leads to a frequent need to remove the protective gear, which involves an increased risk of infection. The multiple steps to remove the suit can take up to 30min (Kitamura, 2014).

The modified *Predicted Heat Strain* (ISO 7933, 2004) model was used to indicate the expected work times (Fig. 1). The estimation was made based on the following assumptions. Standard man was chosen for the model calculations. Medium heavy activity (300W) was taken as the average work rate. The core temperature limit to cease such emergency work was set to 38.5°C. Three clothing types with different moisture permeability (*i*_m_) were selected for comparison: an impermeable outer layer (*i*_m_ = 0.00), a semipermeable outer layer (*i*_m_ = 0.07), and a relatively tight but still permeable outer layer (*i*_m_ = 0.20). The basic clothing insulation in all cases was theoretically taken as 1 clo (0.155 m^2^K W^−1^) for comparative purposes. In all air temperature conditions, the other environmental factors were kept constant. Ambient water vapour pressure was set to 3.0 kPa, air velocity/body motion was 1 m s^−1^, and there was assumed no radiation effect present (work indoors or in shade).

**1:**
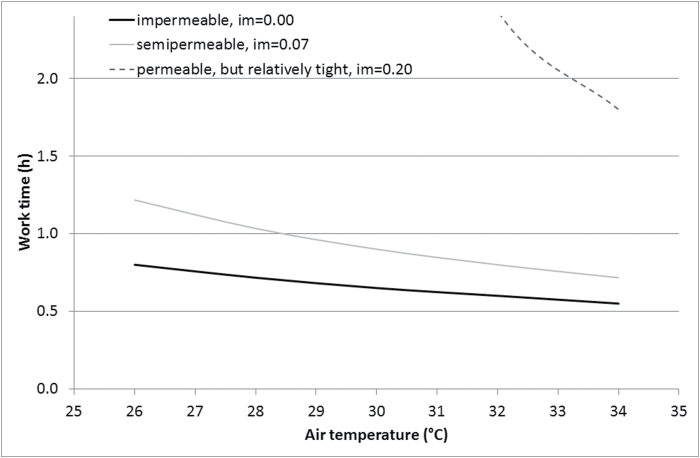
Continuous work times for a work rate of 300W at different air temperatures before reaching a core temperature limit at 38.5°C in clothing with different moisture permeability (*i*_m_).

The chosen work load in impermeable and semipermeable clothing allows 40min or shorter exposure during the hottest periods (Fig. 1) until the core temperature exceeds the suggested safe limit for occupational exposure. Higher core temperature is associated with decreased mental performance and increased misjudgement and mistakes (O’Neal and Bishop, 2010).

Maximizing the moisture permeability and minimizing the clothing layers worn beneath the protective gear, provided that it should be resistant to penetration by body fluids, is a simple way of preventing heat stress and increasing the time spent inside the gear. However, dehydration and water intake must also be considered during extended exposures. A heat stress management program including rehydration should be an essential part of the overall health and safety program in any case.

A desirable addition would be personal cooling used inside the protective clothing, such as cooling vests with ice or phase change materials (PCMs; Gao, 2014) or filtered ventilated coveralls (Kuklane *et al.*, 2012). This may prolong working time to about 2h and reduce the number of gear changes per day. With 2-h work time in protective gear, the number of required personnel could be halved with possible decrease in contaminated waste. The final choice of the cooling method depends on specific air temperature and humidity. Increasing air temperature and, especially, humidity do reduce the effectiveness of air cooling and increase the benefits of PCM products.

The use of PCMs requires freezers or cool areas for solidification after use. Cooling vests with ice are the cheapest and electricity for freezers is required. Power is one of the basic resources to provide healthcare and to cope with epidemics. Otherwise, the other types of PCM, e.g. Glauber’s salt or organic hydrocarbons/wax, with melting/solidifying temperature at about 28°C are available. For workers’ recovery after heat exposure, a room with air temperature below 27°C is recommended. The room or connected facilities could be used for PCM solidification storage. If still unavailable, then the melted PCM can be solidified in a relatively cooler water bath (using underground/well water, etc.), in an underground cave or in a cooler area during night. The higher the melting temperatures are, the less effective cooling is. However, if the temperature gradient is about 6°C or greater, the PCM can still provide a cooling effect.

Considering cooling effect in ventilated garments, the provided air flow should be above 100 l min^−1^. There are filtered fan systems available on the market that manage the flows up to and above 200 l min^−1^ with the battery power lasting at least 5–8h (recharging takes about 2h). Ventilated systems (positive pressure suits) may allow even drinking water in the suit and that may prolong the work time even more.

Table 1 gives a rough cost comparison of the present and a possible future protective clothing system based on 1-day (8-h) shift. It takes into account only the equipment cost. Estimation is based on the work time predictions given in Fig. 1 for the hottest work periods, i.e. 30min for the impermeable set and 2h for the new system that prolongs work period by higher permeability or by use of a cooling device. In both cases, similar final core temperatures are expected to limit the exposure. Also, it is expected that both sets take 30min for dressing, 30min for undressing, and require 30min for recovery between the work periods. As it can be seen the equipment cost of a new, theoretically even a 10 times more expensive solution is almost 3 times higher for a day.

**Table 1.** Comparison of the equipment cost of the present and a possible, 10 times more expensive protective clothing system based on 1-day (8-h) shift. Assumed work time is 30min for present and 2h for the new system. In both cases, expected donning, doffing, and recovery periods are 30min each.

	Present set	New set
Work time under a work session (h)	0.5	2
Workers needed to cover a continuous 8-h work shift (nr.)	4	2
Approximate cost per set ($)	90	900
Number of sets needed per 8-h shift (nr.)	16	4
Total PPE (personal protective equipment) cost per 8-h shift ($)	1440	3600

Simultaneously, there are also other benefits with an actively cooling clothing system. The personnel need to cover one workstation is halved. The personnel have even extra time (about 30min) between the shifts to help with any other tasks or for additional recovery. Due to fewer times of dressing–undressing (16 + 16 times 30min versus 4 + 4 times 30min for present respective new system), there is also less need for assistance and disinfection during these periods. There will be less contaminated waste or fewer amounts of products to be cleaned. The new systems are meant to be reusable (extra costs for decontamination procedures have to be considered) compared to present, supposedly disposable systems, and already 2.5 times reuse will even up the equipment costs at the estimated prices. Infection risks are diminished due to the reduced need for undressing and cleaning procedures.

In conclusion, reducing the risk of infection among the front-line healthcare workers and allowing a doubling of their work capacity could be a critical factor to successfully contain the epidemic. Considering that this epidemic is not the last, and with warmer climate both the epidemics are expected becoming more frequent, and conditions to fight them more severe (IPCC, 2013), then the testing and evaluation for selection of the optimal equipment is required long before missions are set out.
